# Differences in the intestinal microbiome of healthy children and patients with newly diagnosed Crohn’s disease

**DOI:** 10.1038/s41598-019-55290-9

**Published:** 2019-12-11

**Authors:** Kinga Kowalska-Duplaga, Tomasz Gosiewski, Przemysław Kapusta, Agnieszka Sroka-Oleksiak, Andrzej Wędrychowicz, Stanisław Pieczarkowski, Agnieszka H. Ludwig-Słomczyńska, Paweł P. Wołkow, Krzysztof Fyderek

**Affiliations:** 1 Department of Pediatrics, Gastroenterology and Nutrition, Faculty of Medicine, Jagiellonian University Medical College, Wielicka 265, Kraków, 30-663 Poland; 2 Division of Molecular Medical Microbiology, Department of Microbiology, Faculty of Medicine, Jagiellonian University Medical College, Czysta 18, Kraków, 31-121 Poland; 3 Center for Medical Genomics – OMICRON, Jagiellonian University Medical College, Kopernika 7c, Kraków, 31-034 Poland; 4 Division of Mycology, Department of Microbiology, Faculty of Medicine, Jagiellonian University Medical College, Czysta 18, Kraków, 31-121 Poland

**Keywords:** Crohn's disease, Microbiome

## Abstract

The aetiology of inflammatory bowel diseases (IBD) seems to be strongly connected to changes in the enteral microbiome. The dysbiosis pattern seen in Crohn’s disease (CD) differs among published studies depending on patients’ age, disease phenotype and microbiome research methods. The aims was to investigate microbiome in treatment-naive paediatric patients to get an insight into its structure at the early stage of the disease in comparison to healthy. Stool samples were obtained from controls and newly diagnosed patients prior to any intervention. Microbiota was analysed by 16SrRNAnext-generation-sequencing (NGS). Differences in the within-sample phylotype richness and evenness (alpha diversity) were detected between controls and patients. Statistically significant dissimilarities between samples were present for all used metrics. We also found a significant increase in the abundance of OTUs of the *Enterococcus* genus and reduction in, among others, *Bifidobacterium* (*B. adolescentis*), *Roseburia (R.faecis), Faecalibacterium (F. prausnitzii), Gemmiger (G. formicilis), Ruminococcus (R. bromii) and Veillonellaceae (Dialister)*. Moreover, differences in alpha and beta diversities in respect to calprotectin and PCDAI were observed: patients with calprotectin <100 µg/g and with PCDAI below 10 points vs those with calprotectin >100 µg/g and mild (10–27.7 points), moderate (27.5–40 points) or severe (>40 points) CD disease activity had higher richness and diversity of gut microbiota. The results of our study highlight reduced diversity and dysbiosis at the earliest stage of the disease. Microbial imbalance and low abundance of butyrate-producing bacteria, including *Bifidobacterium adolescentis*, may suggest benefits of microbial modification therapy.

## Introduction

Crohn’s disease (CD) belongs to the group of chronic inflammatory bowel disorders (IBD) that may affect any part of the gastrointestinal tract (GI). Intensity and range of lesions may change over time; inflammation usually presents a non-continuous pattern and affects all layers of the intestinal wall. In the past two decades, all developed countries have experienced a steady increase in the prevalence of CD in children^1^, with declining age at presentation and intensifying activity^2^. The underlying cause of this phenomenon is unknown; however, one of the possible reasons is seen in the ongoing civilization changes taking place in the environment. These changes undoubtedly affect the microbiome, which plays important role in the proper functioning of the intestines. With certainty, the microbiome helps to maintain appropriate function of the gut and plays an important role in vitamin production, development of the immune system, epithelial homeostasis and metabolite production, all of which strongly contribute to enhancement of gut barrier function^3–5^. It is difficult to define a healthy gut microbiome as it varies depending on age, environment, or diet, but it should generally be characterized by diversity and stability and fulfil its metabolic function^6^. Numerous studies, both on animal models and humans, have shown that alterations in the gastrointestinal microbiome together with genetic susceptibility and environmental factors are important in initiating the inflammatory process mainly via an abnormal immune response^7–9^.

Exploration of bacterial diversity using culture-independent techniques enabled a better evaluation of the composition of gut microbiota. Changes in the microbiome of CD patients imply both bacterial richness and diversity. The most frequently observed changes are decrease in Bacteroides and Firmicutes and increase in Actinobacteria and Gammaproteobacteria^10^. CD patients’ intestinal microbiome was shown to be poor in *Faecalibacterium prausnitzii*, commensal bacteria with anti-inflammatory properties^11^, *Roseburia intestinalis* and other butyrate-producing bacteria^12^.

So far, only a few studies assessing the microbiome in patients with IBD focused on children. The majority of published papers concerned small cohorts which were heterogeneous in terms of diagnosis and phenotype of IBD, treatment and duration of the disease. Also, the results of two big paediatric CD studies from North America^12^ and Netherland^13^ are not fully compatible.

The aim of this study was to investigate microbiome composition in newly diagnosed, untreated children with CD compared to their healthy peers. We also wanted to assess whether the change in the microbiome correlates with the clinical activity of CD and the biochemical indicators of inflammation.

## Methods

### Patients

#### Ethical approval

All procedures were performed in studies involving human participants were in accordance with the ethical standards of the institutional committee Jagiellonian University Ethics Committee (decision number: 122.6120.68.2015) and with the 1964 Helsinki declaration and its later amendments or comparable ethical standards. All experimental protocols were approved by Jagiellonian University Ethics Committee in Krakow, Poland.

Informed consent was signed by patients’ parents or legal guardians if they are under 18 years of age. If the patient is above 16 years old, then in addition to the consent of the parents or legal guardians, its written consent to participate in the study was also obtained.

This single-centre prospective study was performed at University Children’s Hospital in Kraków, Poland. Newly diagnosed children and adolescents with CD, aged 2 to 18 years, were enrolled in the study group. The diagnosis was based on clinical manifestation, biochemical, endoscopic and histopathological studies, as well as radiological tests in accordance with revised Porto criteria^14^. Morphology and biochemical tests including ESR (erythrocyte sedimentation rate), C-reactive protein (CRP) and faecal calprotectin were checked at admission. The Simple Endoscopic Score for Crohn’s Disease (SES-CD) was assessed during a diagnostic upper gastroscopy and ileocolonoscopy. The phenotype of CD was assessed according to the Paris criteria^15^. Details for Paediatric Crohn’s Disease Activity Index (PCDAI) were collected at admission.

The exclusion criteria were: patients below 2 or above 18 years of age; treatment with antibiotics and/or probiotics during the period of 3 months before collecting the stool sample; confirmed infections of the gastrointestinal tract; isolated perianal disease; and lack of consent to participate in the study. Samples in the study group were collected at admission to the hospital prior to any medical or therapeutic intervention. Faecal calprotectin and all clinical data necessary to assess clinical activity were collected before the commencement of any therapeutic intervention.

The control group consisted of healthy non-hospitalized children (HC – healthy controls) who didn’t meet the exclusion criteria. Their stool samples were collected at home into sterile stool containers, kept refrigerated and transported in cool bags to the laboratory. All stool samples were stored at −80 °C.

### Laboratory procedures

#### DNA extraction from the faecal samples

A detailed DNA isolation protocol was presented in our team’s previous studies^16^. The frozen samples were thawed, precisely weighed (about 0.1 g of stool sample was used) and homogenized in 0.1 ml of saline. After lysis of bacterial and fungal cells with lysozyme (Sigma-Aldrich, Poznań, Poland) (1 mg/ml) and lysostaphin (Sigma-Aldrich, Poznań, Poland) (0.1 mg/ml), samples were incubated at 37 °C for 20 min. Next, 200 μl 75 mM NaOH (Avantor Performance Materials, Gliwice, Poland) was added and samples were incubated at 95 °C for 10 min. After incubation, probes were microcentrifuged (12 000 rpm, 10 min), supernatants were removed, and pellets were resuspended in 500 μl buffer supplemented with β-mercaptoethanol (Sigma-Aldrich, Poznań, Poland). For each sample, lyticase (Sigma-Aldrich, Poznań, Poland) was added (0.1 mg/ml). Probes were incubated at 37 °C for at least 30 min and microcentrifuged (12 000 rpm, 10 min). The next steps of DNA extraction were carried out according to Genomic Mini AX Stool Spin Kit (A&A Biotechnology, Gdańsk, Poland) procedure.

DNA concentration and purity was controlled spectrophotometrically using a NanoDrop apparatus (Thermo Fisher Scientific).

#### 16S library preparation

An amplicon library was created. Amplicons of the selected V3-V4 16 S rRNA gene regions for each sample studied were prepared using following primers with adapters^17^: (F) 5′TCGTCGGCAGCGTCAGATGTGTATAAGAGACAGCCTACGGGNGGCWGCAG 3′ (R)5′ GTCTCGTGGGCTCGGAGATGTGTATAAGAGACAGGACTACHVGGGTATCTAATCC 3′. PCR product was purified using Agencourt AMPure XP (BeckmanCoulterGenomics). Next, library indexing was performed applying Nextera XT Index Kit (Illumina San Diego, California, United States) and one more purification was done. Libraries were fluorescently quantified (Quant-iT™ PicoGreen™ dsDNA Assay Kit - Thermo Fisher Scientific) and normalized to 10 nM and pooled in Eppendorf tube. According to the Illumina protocol for a 2 × 300 cycle run; 10 pM library concentration; 30% PhiX sequencing control V3 for MiSeq high-throughput sequencer were used (Illumina, San Diego, California, United States): http://support.illumina.com/content/dam/illuminasupport/documents/documentation/chemistry_documentation/16s/16s-metagenomic-library-prep-guide-15044223-b.pdf. The sequencing procedure was performed at the Centre for Medical Genomics OMICRON, Jagiellonian University Medical College.

### Sequencing data analysis and statistical analysis

The samples were processed and analysed using the Quantitative Insights Into Microbial Ecology (QIIME2, version 2018.11)^18^ custom pipeline. All following procedures in this section were conducted in the QIIME2 environment (using QIIME2 plugins). Demultiplexed paired-end reads from MiSeq (2 × 300 bp) were trimmed to remove primers and poor quality bases with Cutadapt^19^. The trimmed sequences were denoised and joined with DADA2^20^. Then open-reference clustering of features^21^ and reference-based chimera filtering were performed using vsearch^22^ and the Greengenes database at 99% similarity^23^. The generated operational taxonomic units (OTUs) were assigned to taxonomy using a pre-trained Naive Bayes classifier^24^.

The classifier was trained on the region of the target sequences that were sequenced. Briefly, we extracted sequences from Greengenes 13.8, 99% OTUs with locus-specific sequences from V3-V4 Illumina 16 S Amplicon Primers. Based on the taxonomy generated, we filtered our feature-table to include only assigned reads of the kingdom *Bacteria* and to remove singleton features and elements with fewer than 10 reads. The filtered feature-table was used to generate the tree for phylogenetic diversity analyses. Briefly, we *de novo* aligned sequences using MAFFT^25^ and masked highly variable positions^26^. Then, we applied FastTree^27^ to generate an unrooted phylogenetic tree from masked alignment. In the final step, we rooted the tree at the midpoint of the longest tip-to-tip distance in the unrooted tree. Rarefaction curve analysis of the data obtained was used to estimate the completeness of microbial communities sampling. Subsequently, we computed several alpha (Shannon’s diversity index, observed OTUs, Faith’s Phylogenetic Diversity, Pielou’s evenness) and beta diversity metrics (Jaccard distance, Bray–Curtis distance, unweighted UniFrac distance, weighted UniFrac distance and generated principal coordinates analysis PCoA) plot using Emperor^28^ for each of the beta diversity metrics. Group significance between alpha and beta diversity indexes was calculated with QIIME2 plugins using the Kruskal–Wallis test and permutational multivariate analysis of variance (PERMANOVA), respectively. Correlations with alpha diversity indexes were calculated with QIIME2 plugin using the Spearman correlation. Differential abundance between groups on each taxonomic level was tested with ANCOM^29^, with a taxa-wise correction for multiple testing. The ANCOM procedure compares the relative abundance of a taxon between two groups, by performing statistical tests on data transformed by an additive log-ratio (Aitchison’s log-ratio) of the abundance of given taxon versus the abundance of all other taxa, individually. The W-value generated by ANCOM method is a count of a number of sub-hypotheses (Aitchison’s log-ratio) that were detected to be significantly different across tested groups for a given taxon. The significance of a test for a given taxon is determined by the Benjamini-Hochberg correction that controls for False Discovery Rate (FDR) at 0.05.

## Results

A total of 82 children and adolescents aged 2–18 years (mean 148.52 months; SD = 41.09) were included in the study. General characteristics of participants are given in Table 1. None of the patients presented with isolated L4a or L4b location or exclusive perianal disease. In two-thirds of the patients, initial disease activity (according to PCDAI) was assessed as mild to moderate. The endoscopic score (SES-CD) was 17.3 (SD = 8.04).

**Table 1:** General characteristics of participants.

	Crohn’s disease patients (CD) (n = 64)	Healthy controls (HC) (n = 18)
Sex: male	61% (39/64)	44% (8/18)
Age (months; SD)	151.41 (SD = 42.41)	138.28 (SD = 35.16)
Height (cm; SD)	150.77 (SD = 20.6)	146.67 (SD = 20.52)
Weight (kg, SD)	38.32 (SD = 13.79)	41.75 (SD = 17.37)
BMI	16.24 (SD = 2.65)	18.3 (SD = 3.49)
**Disease distribution by Paris classification: (number of patients)**		
A1a	13	
A1b	47	
A2	4	
L1	2	
L2	8	
L3	15	
L4aL1	10	
L4aL2	11	
L4aL3	17	
B1	41	
B2	20	
B2 B3	3	
G0	40	
G1	24	
p	1	
PCDAI:	31.48 (SD = 15.44)	
≤40 (mild and moderate)	43	
>40 (severe)	21	

CD – Crohn’s disease, m – months, SD – standard deviation, PCDAI – Paediatric Crohn’s Disease Activity Index. Paris classification: age: A1a: 0–10 y, A1b: 10–17 y, A2: 17–40 y; location: L1 – distal 1/3 ileum and/or limited cecal disease; L2 – colonic; L3 – ileocolonic; L4a – upper disease proximal to ligament of Treitz; L4b – upper disease distal to ligament of Treitz and proximal to distal 1/3 ileum, behaviour: B1 – non-structuring non-penetrating; B2 – structuring; B3 – penetrating; B2B3 – both penetrating and structuring disease either at the same or different times; p – perianal disease modifier; growth: G0 – no evidence; G1 – growth delay.

### Quality of DNA isolates

Isolates with a concentration of >100 ng/µl and a purity ratio A_260_/A_280_ > 1.7 were used to prepare libraries and sequencing.

### 16 S rRNA V3-V4 region sequencing results

After clustering, removal of chimeras and filtering, 2,008,883 sequences from 82 samples with 619 OTUs were obtained. Percentage of annotated OTUs on the phylum (L2), class (L3), order (L4), family (L5), genus (L6), and species (L7) level 100%, 100%, 98.8%, 93.7%, 72.2% and 24.9% OTUs, respectively. The median frequency was 23,662 with IQR 15,919–31,744. Based on the rarefaction curve, the alpha and beta diversity metrics were calculated on a rarefied frequency-feature table with a minimum number of 4252 sequences per sample. One sample was excluded from diversity analyses due to low read count.

**Figure 1:**
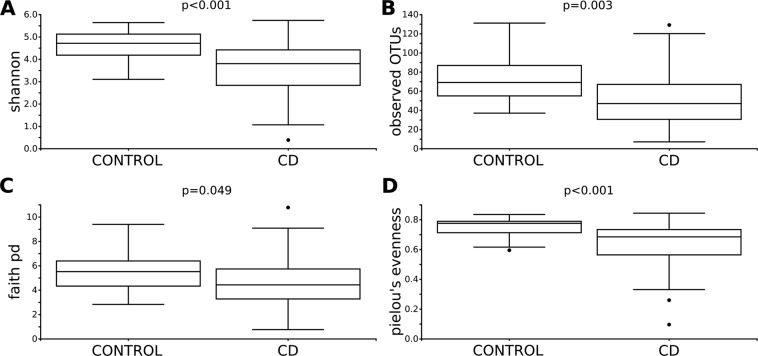
Alpha diversity analysis of control and CD patients. Within-sample diversity measured by Shannon index (**A**), observed OTUs (**B**), Faith’s phylogenetic diversity (**C**) and Pielou’s measure of species evenness (**D**). Kruskal-Wallis test was performed to analyse statistical significance.

Differences in the within-sample phylotype richness and evenness (alpha diversity) were detected between controls and CD patients. Shannon’s diversity index (p < 0.001), observed OTUs (p = 0.003), Faith’s Phylogenetic Diversity (p = 0.049) and Pielou’s evenness (p < 0.001), were statistically different between controls and CD patients (Fig. 1).

**Figure 2:**
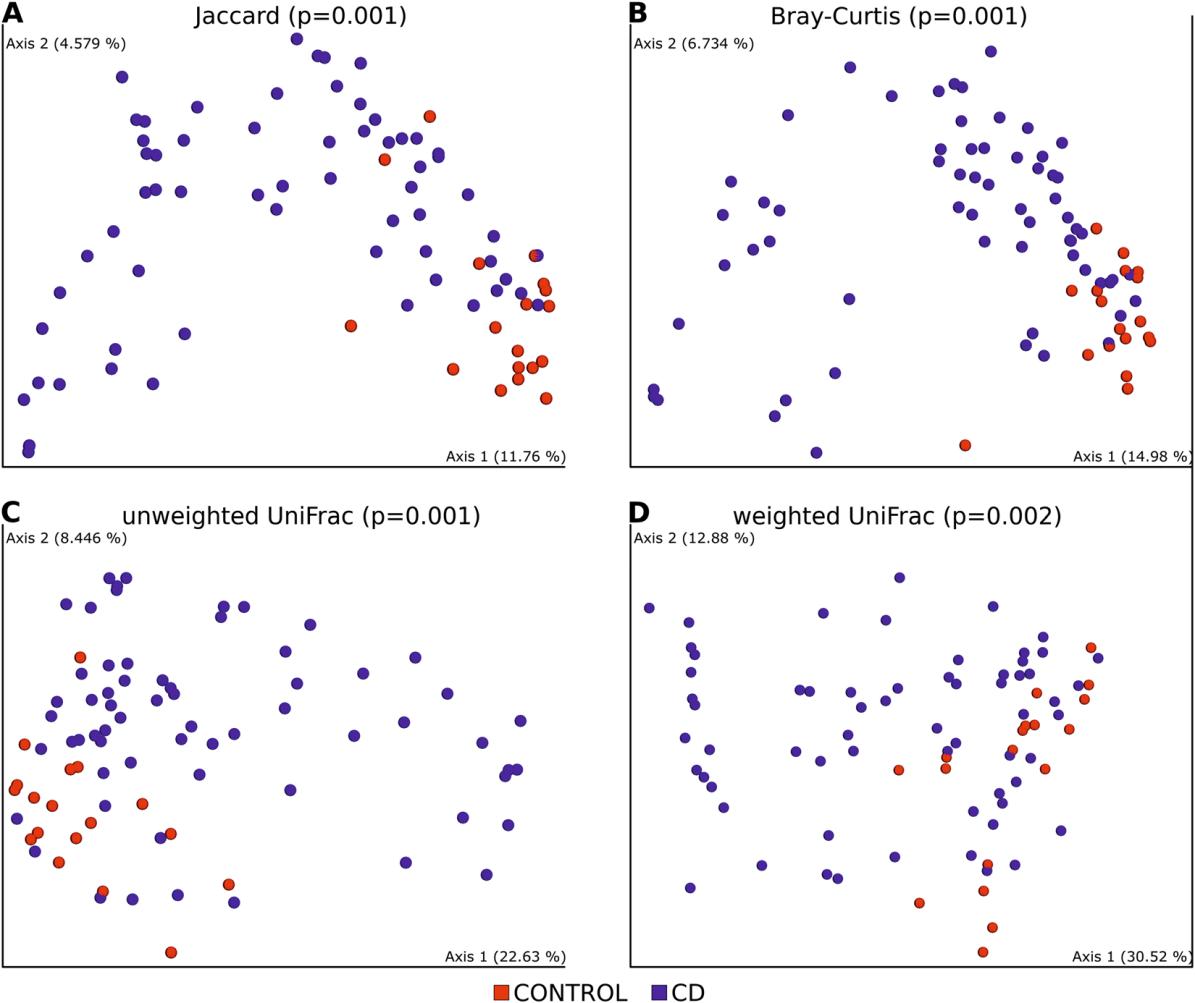
PCoA 2D plots of beta diversity analysis of control and CD patients. Between-sample dissimilarities were measured by Jaccard distances (**A**), Bray-Curtis distance (**B**), unweighted UniFrac distances (**C**) and weighted UniFrac distances (**D**). Permutational multivariate analysis of variance (PERMANOVA) was performed to analyse statistical significance.

Statistically significant dissimilarities between samples (beta diversity) were seen in all distance metrics: Jaccard distance (p = 0.001), Bray–Curtis distance (p = 0.001) unweighted UniFrac distance (p = 0.001), weighted UniFrac distance (p = 0.002). PCoA 2D plots from beta diversities are presented in Fig. 2.

Relative abundance of the top bacteria at phylum level (L2) in control and CD groups were: Firmicutes (77.03% vs 68.75%), Actinobacteria (17.17% vs 20.61%), Bacteroidetes (2.74% vs 4.01%), Verrucomicrobia (2.12% vs 3.56%) and Proteobacteria (0.88% vs 2.63%), respectively.

Top bacteria at class level (L3) in control and CD groups were: Clostridia (71.22% vs 36.80%), Bacilli (3.74% vs 25.91%), Actinobacteria (13.77% vs 16.15%), Erysipelotrichia (2.08% vs 6.03%), Coriobacteriia (3.41% vs 4.45%), Bacteroidia (2.74% vs 4.01%), Verrucomicrobiae (2.12% vs 3.56%) and Gammaproteobacteria (0.88% vs 2.49%), respectively.

**Figure 3:**
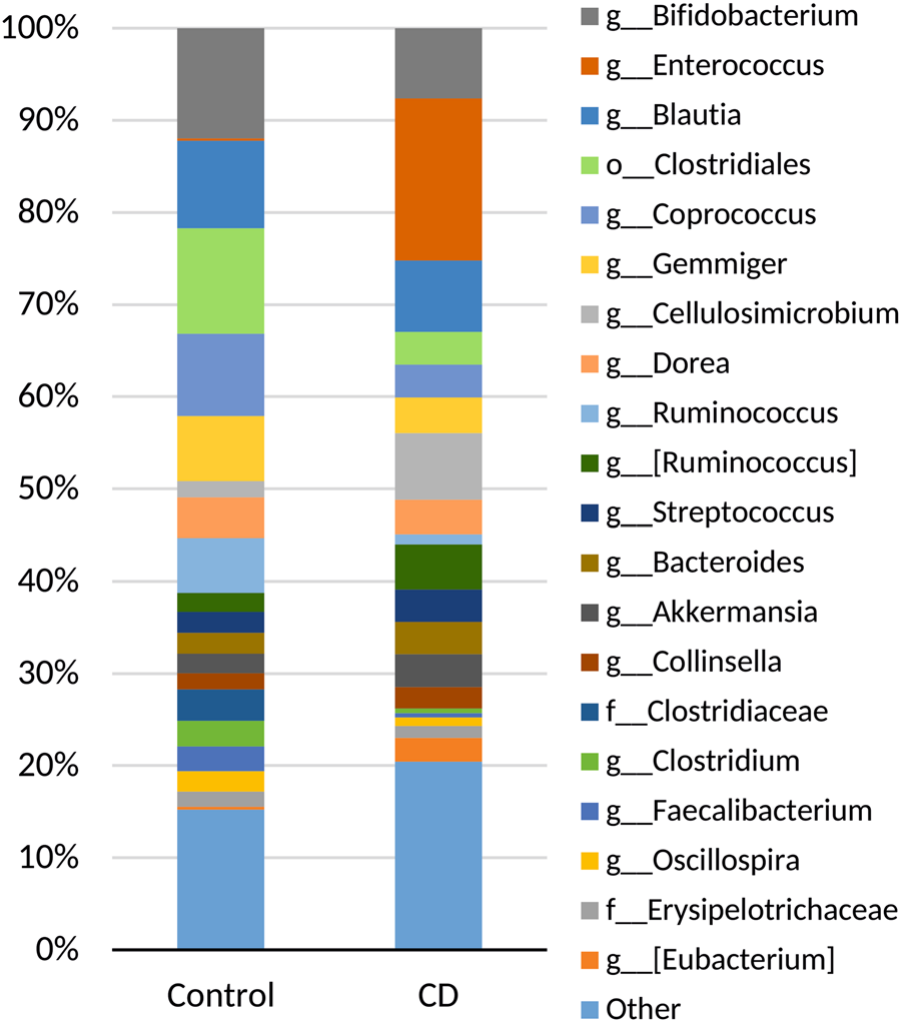
Composition of the bacterial community at the genus (L6) level for control and CD samples. Relative abundance of top 20 genera are shown.

Composition of the bacterial community at the genus level (L6) for control and CD samples are shown in Fig. 3.

**Figure 4:**
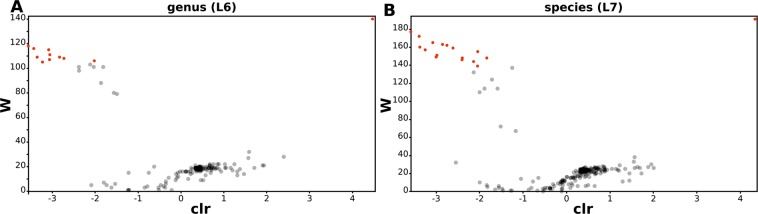
Volcano plots of differentially expressed OTUs between control and CD patients at genus (**A**) and species (**B**) levels. ANCOM analysis was performed to analyse a statistical significance. Statistically significant OTUs are represented as red rounds.

ANCOM analysis at the genus (L6) and species (L7) levels showed that there are 11 genera (Table 2 and Fig. 4A) and 17 species (Table 3 and Fig. 4B) differently expressed between controls and CD patients. The analytical method allowed to determine the exact species names for only 6 of them. We have found a significant increase in the abundance of OTUs of the genus *Enterococcus* and a statistically significant reduction in *Bifidobacterium* (*B. adolescentis*), *Adlercreutzia*, *Clostridium* (*C. celatum*), *Coprococcus, Roseburia (R. faecis), Faecalibacterium (F. prausnitzii), Gemmiger (G. formicilis), Ruminococcus (R. bromii) and Veillonellaceae (Dialister)*.

**Table 2:** Statistically significant ANCOM results at genus level. Relative abundance across all samples or features within a group were summed.

	Control	CD	W-statistic value	clr mean difference
*g__Adlercreutzia*	0.19%	0.04%	106	−2.02
*g__Enterococcus*	0.29%	17.62%	140	4.47
*o__*Clostridiales;__;__	11.48%	3.55%	109	−3.35
*f__Clostridiaceae;g__*	3.37%	0.03%	118	−3.57
*g__Clostridium*	2.80%	0.50%	111	−3.05
f__Lachnospiraceae;__	1.10%	0.58%	108	−2.72
*g__Coprococcus*	8.93%	3.55%	107	−3.06
*g__Roseburia*	1.94%	0.29%	116	−3.43
*g__Faecalibacterium*	2,67%	0,49%	109	−2.83
*g__Gemmiger*	7.03%	3.88%	105	−3.23
*g__Ruminococcus*	5,94%	1,06%	115	−3.08

clr - centered log-ratio.

**Table 3:** Statistically significant ANCOM results at species level. Relative abundance across all samples or features within a group were summed.

	CONTROL	CD	W-statistic value	clr mean difference
*Bifidobacterium adolescentis*	6.56%	0.67%	151	−2.98
*f__*Coriobacteriaceae;g__;s__	0.70%	0.90%	146	−2.40
*g__Adlercreutzia;s__*	0.19%	0.04%	155	−2.04
*g__Enterococcus;s__*	0.29%	17.45%	191	4.34
o__Clostridiales;__;__;__	0.86%	0.068%	160	−3.37
f__ Clostridiaceae;g__;s__	3.37%	0.026%	177	−3.589
*g__Clostridium;s__*	0.578%	0.14%	139	−2.04
*Clostridium celatum*	1.42%	0.13%	159	−2.61
f__Lachnospiraceae;__;__	1.10%	0.58%	162	−2.75
f__Lachnospiraceae;g__;s__	1.55%	0.10%	144	−2.13
*g__Anaerostipes;s__*	0.22%	0.06%	148	−1.83
*g__Coprococcus;s__*	8.61%	3.47%	149	−3.00
*Roseburia faecis*	1.89%	0.27%	172	−3.39
*Faecalibacterium prausnitzii*	2.67%	0.49%	163	−2.85
*Gemmiger formicilis*	7.03%	3.89%	157	−3.25
*Ruminococcus bromii*	4.80%	0.62%	165	−3.07
g__*Dialister;s__*	0.92%	0.35%	148	−2.39

clr - centered log-ratio.

### Correlation of microbiome structure and clinical parameters

**Figure 5:**
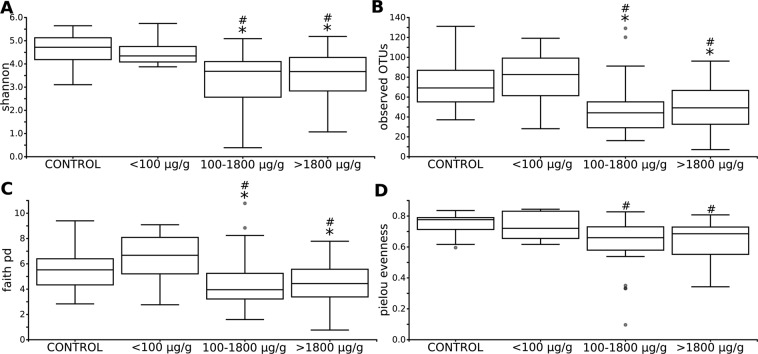
Alpha diversity analysis of control and CD patients with different level of calprotectin. Within-sample diversity measured by Shannon index (**A**), observed OTUs (**B**), Faith’s phylogenetic diversity (**C**) and Pielou’s measure of species evenness (**D**). Kruskal-Wallis with Post-hoc was performed to analyse statistical significance. Statistically significant values between control and other groups were represented as “#”. Statistically significant values between PCDAI < 10 group and with other groups: mild (10–27.5 points; 21 patients), moderate (>27.5–39 points, 17 patients) and severe (>40 points; 21 patients) CD disease activity (Supplementary Fig. S1 online) were represented as “*”.

**Figure 6:**
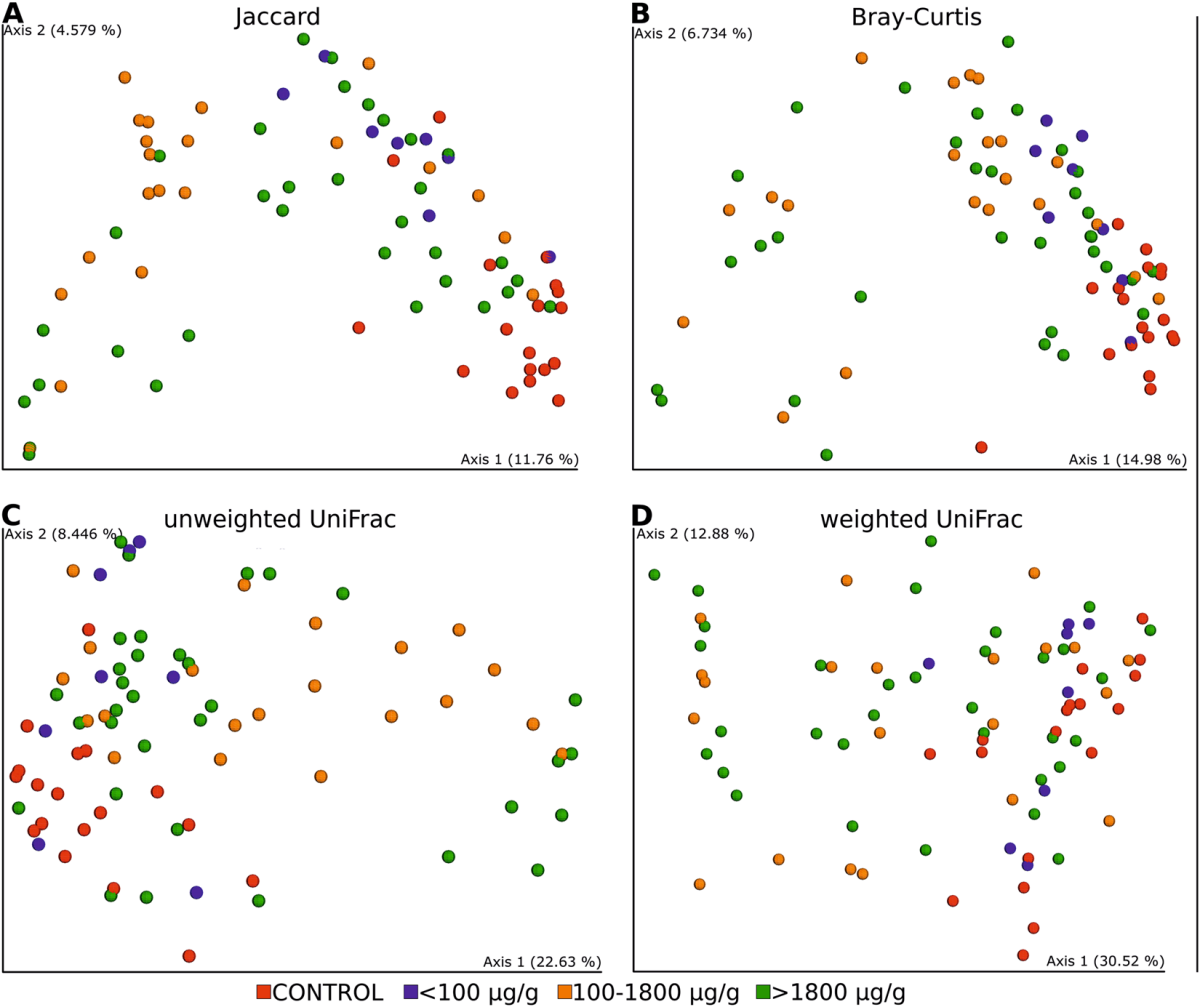
PCoA 2D plots of beta diversity analysis of control and CD patients with different level of calprotectin. Between-sample dissimilarities were measured by Jaccard distances (**A**), Bray-Curtis distance (**B**), unweighted UniFrac distances (**C**) and weighted UniFrac distances (**D**).

Alpha and beta diversity does not correlate with most of the clinical parameters. We did not find associations between microbiome structure and Paris scale parameters, SES-CD and PCDAI or infectious biochemical parameters like ESR and CRP, serum iron and albumin level. However, we have observed differences in alpha (Fig. 5) and beta diversities (Fig. 6) with respect to calprotectin: patients with calprotectin <100 µg/g (n = 8) have higher richness and diversity of gut microbiota than patients with calprotectin levels between 100 µg/g and 1800 µg/g (n = 21) and above 1800 µg/g (n = 33). We also observed some microbiome change associations with PCDAI of less than

10 points (5 patients) vs those with mild (10–27.5 points; 21 patients), moderate (>27.5–39 points, 17 patients) and severe (>40 points; 21 patients) CD disease activity (Supplementary Fig. S1 online).

## Discussion

In our cohort of treatment-naïve CD patients, a reduction in microbiota diversity and richness compared to healthy controls was shown. We found that 11 genera and 17 species differed significantly between CD patients and HC. The respective alteration was observed in OTUs belonging to *Actinobacteria* and *Firmicutes*.

A significant increase in the abundance of OTUs of the genus *Enterococcus* in our paediatric CD patients was consistent with the findings of other authors^30,31^. In our cohort, we also found a significant reduction in the abundance of OTUs of the genus (and species): *Bifidobacterium* (*B. adolescentis*), *Adlercreutzia*, *Clostridium* (*C. celatum*), *Coprococcus, Roseburia (R. faecis), Faecalibacterium (F. prausnitzii), Gemmiger (G. formicilis), Ruminococcus (R. bromii) and Veillonellaceae (Dialister)*. Similar observations, i.e. a gain in *Enterococcus* and significant decrease in bacteria producing short-chain fatty acids (SCFA) like *Coprococcus*, *Faecalibacterium*, *Roseburia*, *Ruminococcus*, *Anaerostipes*, *Blautia*, *Lachnospira* and *Sutterella* were reported by others, both in paediatric^12,32–34^ as well as adult^9,35^ CD patients.

Although numerous studies confirm that microbiome in IBD patients is different in terms of quantity and quality compared to healthy people, based on the research carried out so far, it has not been possible to determine a single strain of bacterial species responsible for causing inflammation associated with CD. Probably, a widely understood change in the microbiome at various taxonomic levels and also in a different anatomic location in the GI tract plays a pathogenic role. Similar to our observation, in adult CD patients, Joossens *et al*. reported a decrease in *Faecalibacterium prausnitzii*, *Bifidobacterium adolescentis*, *Dialister* and did not characterize *Clostridium* species, and there was an increase in *Ruminococcus gnavus*. Their study cohort consisted of already diagnosed and treated patients^35^. The presence of similar changes in the bacterial flora in children and adults indicates the existence of a definite age-independent pattern for changing the microbiome in CD patients. What is more, the dysbiosis existing in treated patients may suggest ongoing inflammation and incomplete response to the treatment or shows that this type of dysbiosis is permanent and not related to the treatment or illness activity. In the latter case, bacteria are rather indicators than mediators of IBD. This is in concordance with the observation of Vrakas *et al*., who found changes in the microbiome characteristic for CD in both active as in-active individuals^36^. On the other hand, O’Brien *et al*. didn’t find any imbalance or reduced microbial diversity in patients whose only endoscopic lesions were aphthous ulcers. The authors suggest that dysbiosis improves when patients are in remission^37^.

Results of published studies are inconsistent and difficult to compare as the research differs not only in terms of selection of the study groups, the age of patients, disease duration and methods of treatment, but also the methodology applied. Depending on study design, luminal (stool samples)^9,32,33,35,38,39^ or/and mucosal (biopsy)^12,36,37,40,41^ microbial environment were investigated in IBD patients. Prevalence of one material over another is under discussion, although it seems that mucosal biopsy gives deeper insight into microbial alteration^42^. On the other hand, obtaining stool samples is easier, non-invasive and repeatable even in short periods of time.

Studies on the microbiome in children are still relatively few and most often concern small groups of patients. In the big cohort study of treatment-naïve new-onset paediatric CD patients, the imbalance was shown only in the microbiome obtained from biopsy, not from stool samples^12^. Also, Douglas *et al*. state that microbiome from stool samples differs drastically when compared with mucosa samples^41^. In contrast, our results are in agreement with the results of those authors who were able to confirm imbalance both in numbers as well as in biodiversity in faecal microbiome^13,33^. In our study, we assessed a quite large, homogeneous group of patients and we decided to use a non-invasive method (stool samples), which allowed us to include fully healthy children who did not require a colonoscopy to HC group. Regardless of whether the microbiome is assessed in stool or biopsy samples, the results obtained by different authors show some differences. For example, what is intriguing, Hansen *et al*., in biopsies, showed an increase in *F. prausnitzii* in de-novo diagnosed paediatric CD^40^. Generally *F. prausnitzii* is an essential component of the intestinal microbiome of healthy people. Numerous studies indicate that this bacterium can be considered a microbiological marker of inflammation in the gastrointestinal tract^43,44^. We, as many authors mentioned, confirmed lack of *F. prausnitzii* in the stool of CD patients. The question is whether the reduction in *F. prausnitzii* in the stool may precede its reduction in the mucous membrane, the natural habitat for this gram-positive, anaerobic species, or conflicting results follow the method used. Perhaps to answer this question it would be necessary to compare the biopsy results with those of stool in the same patient group.Another study indicates that IBD dysbiosis was mainly characterized by decreased abundance of *Alistipes finegoldii* and *Alistipes putredinis*, members of the core microbiota of healthy state. The authors did not observe large differences in the abundance of *F. prausnitzii*^13^. In our study, we have found a statistically significant decline in *F. prausnitzii* abundance while *Alistipes finegoldii* and *Alistipes putredinis*, although present, did not show significant differences between disease and healthy state. In general, however, we support the authors’ opinion that IBD microbiome is characterized more by diminishing abundance of certain bacterial species than by an increase in pathogens. In the study conducted by Kugathasan *et al*., 14 genera associated with CD were identified with the largest increase in *Aggregatibacter* and the greatest decrease in *Roseburia* and SCFA-producing bacteria^34^. Gevers *et al*. underline the role of *Fusobacterium* as a biomarker of inflammation in IBD^12^ and Douglas *et al*. states that *Desuflovibrio* and *Akkermansia muciniphila* are the IBD most informative genera^41^. Our results support the theory that lack of *F. prausnitzii* and *B. adolescentis* in the stool can serve, although not always with statistical significance, as a biomarker signal of dysbiosis typical for CD^13,33^. Pascal *et al*. have attempted to establish microbial biomarkers of CD and the results obtained by them give hope for the creation of a promising non-invasive diagnostic method in CD suspected patients with nonspecific signs of disease^39^. We have also observed a decrease in the abundance of *Roseburia* in newly diagnosed children, which may contribute to the suggestion of Imhann *et al*. that these changes can precede the onset of IBD^9^.

Environment, diet and genetic background seem to be key players in the creation of gut microbiota. Ashton *et al*. observed compositional changes in microbiota between individual patients as well as differences between patients and healthy controls^45^. Interestingly, the microbiome in untreated patients and their healthy siblings showed some similarity which may confirm the role of environment and dietary habits in microbiome composition. On the other hand, Ijaz *et al*. showed that the microbiome in healthy adult relatives of paediatric CD patients is more similar to the microbiome in healthy adult controls^46^. Joossens *et al*. did not reveal the same type of dysbiosis as in CD patients either in unaffected relatives or healthy controls^35^. Those two studies may confirm that alteration in microbiome composition is related more strongly to the illness itself than to genetic background and nutritional habits or is related to some other environmental factors.

Assessment of microbiome composition in treatment-naïve paediatric population gives an opportunity to determine changes unaffected by treatment and accompanying health problems. One of the purposes of our study was to find out the correlation between microbiome, clinical activity and manifestation of CD and the biochemical indicators of inflammation. Kugathasan *et al*. have attempted to attribute changes in the microbiome to the location of illness in the GI tract and typical complications, i.e. stenosis and fissures. They suggest a correlation between *Ruminococcus* with structuring complication and *Veillonella* with penetrating form^34^. As for localization, Pascal *et al*. detected increased abundance of *Enterococcus faecalis* when CD was localized in ileum compared with ileocolon location^39^. We did not find a similar relationship in our patients.

The study investigating microbiome in treatment-naïve new-onset paediatric CD patients confirmed that alteration in abundance of several taxa and diminished richness of taxa was related to the intensity of inflammation^12^. We have also observed a decrease in alpha and beta diversities with an increased level of faecal calprotectin and higher disease activity index (PCDAI). This indicates a link between dysbiosis and inflammation of the gastrointestinal tract. The question is whether dysbiosis is the direct cause of inflammation or rather its consequence. Perhaps, apart from the trigger factor, the mechanism of a vicious circle works: persistent inflammation deepens dysbiosis and vice versa. Similarly to other authors, we show no change in the diversity of the microbiome depending on other disease activity and biochemical markers (CRP, ESR)^9^. Those parameters were not statistically significantly associated with changes in microbiome. This in turn may indicate that, taking into account microbiom, local inflammation (i.e. located in the gastrointestinal tract and reflected by high calprotectin level) is more important than generalised inflammatory status.

The strengths of this study are a homogeneous group of patients, consistent data and sample collection. The main weakness, in our opinion, is the lack of mucosa biopsies for assessment of mucosa-associated microbiome. Additional study limitation is lack of a mock-community or spike-in positive control, which could accurately characterized performance of our metagenomic workflow, and could be used as a reliable reference against which to standardize sample between different experiments and studies.

## Conclusion

Our work contributes to the data helping understand intestinal dysbiosis in new-onset, treatment-naive paediatric CD patients.

If the change in intestinal microflora is one of the risk factors for the development and / or persistence of inflammation in IBD, then the microbiome-oriented treatment should be a component of the therapeutic goals. Such treatment methods as antibiotics, pro- and prebiotics as well as faecal microbiota transplant can be taken into account. Furthermore, considering the relationship between microbiota and genetic predisposition, the usefulness of a microbiome-based preventive treatment in high-risk groups may be considered.

Consistent data confirming low abundance and diversity of *Bifidobacterium* in CD patients suggest that further studies are needed to answer the question whether it is possible to use *Bifidobacterium adolescentis* as a probiotic.

Although studies of the faecal stream may face limitations in detecting microbes associated with the mucus layer and, thus, those more directly involved in disease initiation and perpetuation, the application of this method is far less invasive and allows multiple and reproducible material collection which makes it possible to monitor disease progression.

It is still an open field and active research area for investigation of the best therapeutic strategies to successfully manipulate microbiota in CD patients.

## Supplementary information

Alpha diversity analysis of control and CD patients depending on the PCDAI scale.
