# Supplementary material for: Differences in the intestinal microbiome of healthy children and patients with newly diagnosed Crohn’s disease
Source: Sci Rep. 2019 Dec 11;9:18880. doi: 10.1038/s41598-019-55290-9 (PMC6906406; doi:10.1038/s41598-019-55290-9)
Supplement: Supplementary file 1 — Alpha diversity analysis of control and CD patients depending on the PCDAI scale. [file 41598_2019_55290_MOESM1_ESM.pdf]

Differences in the intestinal microbiome of healthy children and patients with newly diagnosed Crohn's disease

Kinga Kowalska-Duplaga[1], Tomasz Gosiewski[2], Przemysław Kapusta[3], Agnieszka Sroka-Oleksiak[2,4], Andrzej Wędrychowicz[1], Stanisław Pieczarkowski[1], Agnieszka H. Ludwig-Słomczyńska[3], Paweł P. Wołkow[3], Krzysztof Fyderek[1].

[1]Department of Pediatrics, Gastroenterology and Nutrition, Faculty of Medicine, Jagiellonian University Medical College, Kraków, Poland

[2]Division of Molecular Medical Microbiology, Department of Microbiology, Faculty of Medicine, Jagiellonian University Medical College, Kraków, Poland

[3]Center for Medical Genomics – OMICRON, Jagiellonian University Medical College, Kraków, Poland

[4]Division of Mycology, Department of Microbiology, Faculty of Medicine, Jagiellonian University Medical College, Kraków, Poland

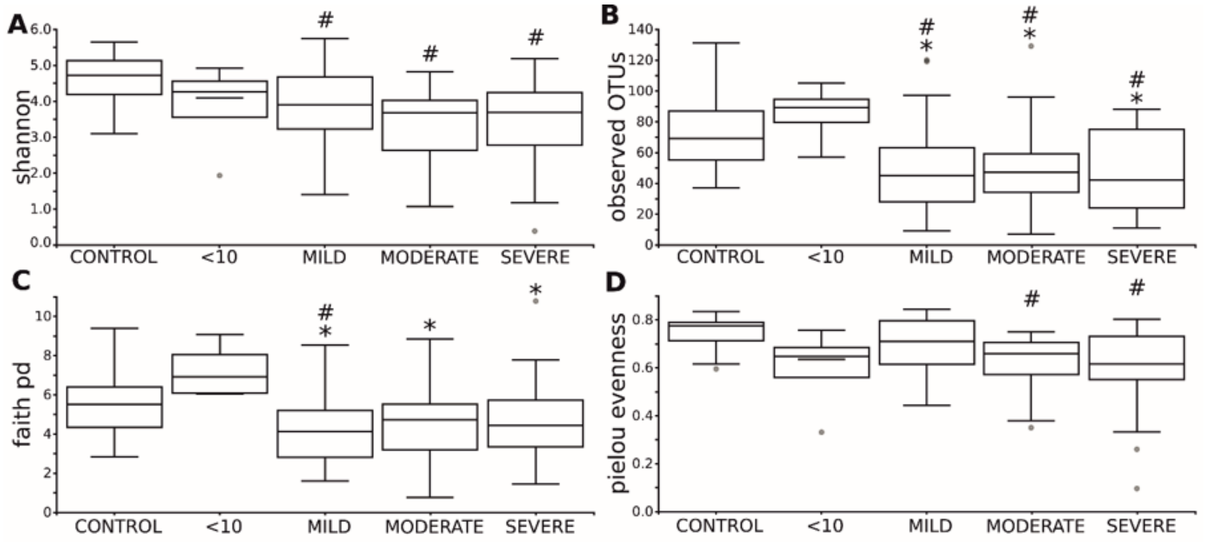

Supplement 1. Alpha diversity analysis of control and CD patients depending on the PCDAI scale.

Figure legend:

Supplementary Figure S1 . Alpha diversity analysis of control and CD patients depending on the PCDAI scale. Within-sample diversity measured by Shannon index (A), observed OTUs (B), Faith's phylogenetic diversity (C) and Pielou's measure of species evenness (D). Kruskal-Wallis with Post-hoc was performed to analyse statistical significance. Statistically significant values between control and other groups were represented as "#".Statistically significant values between PCDAI <10 group and with other groups: mild (10–27.5 points; 21 patients), moderate (>27.5–39 points, 17 patients) and severe (>40 points; 21 patients) CD disease activity (Supplementary Figure S1 online) were represented as "*".
